# Exosomes and Brain Metastases: A Review on Their Role and Potential Applications

**DOI:** 10.3390/ijms221910899

**Published:** 2021-10-08

**Authors:** Filipa D. Oliveira, Miguel A. R. B. Castanho, Vera Neves

**Affiliations:** Instituto de Medicina Molecular, Faculdade de Medicina, Universidade de Lisboa, Av. Professor Egas Moniz, 1649-028 Lisboa, Portugal; fdoliveira@medicina.ulisboa.pt (F.D.O.); macastanho@medicina.ulisboa.pt (M.A.R.B.C.)

**Keywords:** exosomes, tumor microenvironment, pre-metastatic niche, drug delivery system, metastatic cancer, brain metastases, blood–brain barrier

## Abstract

Brain metastases (BM) are a frequent complication in patients with advanced stages of cancer, associated with impairment of the neurological function, quality of life, prognosis, and survival. BM treatment consists of a combination of the available cancer therapies, such as surgery, radiotherapy, chemotherapy, immunotherapy and targeted therapies. Even so, cancer patients with BM are still linked to poor prognosis, with overall survival being reported as 12 months or less. Intercellular communication has a pivotal role in the development of metastases, therefore, it has been extensively studied not only to better understand the metastization process, but also to further develop new therapeutic strategies. Exosomes have emerged as key players in intercellular communication being potential therapeutic targets, drug delivery systems (DDS) or biomarkers. In this Review, we focus on the role of these extracellular vesicles (EVs) in BM formation and their promising application in the development of new BM therapeutic strategies.

## 1. Introduction

Cancer is amongst the leading causes of death worldwide, causing nearly 10 million deaths in 2020, while metastases are the primary cause of cancer-related death [1]. Brain metastases (BM), in particular, are a frequent complication in patients with advanced cancer, with critical impact on neurological function, quality of life, prognosis and survival [2,3]. The types of cancer most frequently associated to brain metastases are lung, breast and skin (melanoma) [3,4] with lung and breast cancers being the two most frequently diagnosed cancers in the world [1]. The increase in BM incidence can be in part explained by improvement of systemic cancer treatment, which increases patients’ life span. In addition, better imaging technology allows earlier cancer detection. However, BM treatment is complex, and may include one or a combination of available therapies that include surgery, radiotherapy, chemotherapy, immunotherapy and targeted therapies [3,4,5]. Nonetheless, the prognosis for cancer patients with BM remains poor, with the majority of the results from clinical trials showing patients’ overall survival below 12 months [5].

One of the major obstacles to develop an effective BM treatment relies on the impermeable nature of the blood–brain barrier (BBB), which confers the brain a sanctuary status, where metastatic cancer cells can settle and proliferate as they are protected from most anticancer drugs [4,6]. In this context, the BBB is nowadays one of the most extensively studied biological barriers for which there is a need to understand its complex physiology and develop new strategies to effectively deliver anticancer drugs to the brain [7]. Exosomes, nano-sized extracellular vesicles with a natural ability to cross the BBB, have been simultaneously described as a strategy used by cancer cells to promote the metastization process and as a promising drug delivery system (DDS) for BM targeting. In this Review, we will focus the role of exosomes in BM formation and discuss their impact on metastatic cancer treatment.

## 2. Exosomes and Their Role in Cancer and Brain Metastases

### 2.1. Exosomes’ Biogenesis and Composition

Exosomes are nano-sized (30–150 nm) extracellular vesicles (EVs) formed by a lipid bilayer surrounding an organelle-deprived cytosol with several biomolecules, such as proteins, glycans, lipids, RNA and DNA [8,9]. These vesicles are naturally found in various body fluids, such as blood plasma and urine, and they are produced by most (if not all) cells [10]. Exosomes’ biogenesis starts with the invagination of the endosomal limiting membranes resulting in intraluminal vesicles (ILVs) inside the endosome, forming the so-called multivesicular body (MVB) [9,11]. This MVB will then fuse with the cell membrane, releasing the ILVs which are now referred to as exosomes [9]. Therefore, contrary to other types of EVs that result from direct plasma membrane budding, exosomes come from the endocytic pathway [9]. In addition to the site of the biogenesis, another feature that distinguishes exosomes from other types of EVs is the size. EVs produced by the cell from direct budding of the plasma membrane are considered to be larger than exosomes (100–1000 nm) [11,12]. Exosomes’ production is a complex process which involves several different protein machineries, namely: (1) endosomal sorting complexes required for transport (ESCRT) to regulate ILV formation; (2) tetraspanins, transmembrane proteins which enable vesicle formation by promoting membrane curvature; (3) Rab GTPase proteins for endosomal trafficking control and (4) several enzymes, such as sphingomyelinase, to produce ceramides and promote vesicle formation [9,11,12]. Interestingly, some of these protein networks and complexes are also involved in the formation of other types of EVs and, therefore, despite having distinctive sites for biogenesis and sizes, exosomes and other EVs share common protein machinery [9,12]. Importantly, the need for such a complex protein machinery for exosome formation implies that this is a heavily regulated process involving the secretion of specific substrates, which suggest a critical function for these vesicles, as discussed in the next section. Another piece of evidence of the specificity and selectivity of exosomes’ biogenesis is the heterogeneity of the exosomes’ population with different subpopulations exhibiting different molecular compositions and organ distribution, and suggesting different roles [13]. Recent studies suggest the classification of the subpopulations of exosomes as follows: Exo-Large (90–120 nm), Exo-Small (60–80 nm) and membrane-less exomeres (<50 nm) [13].

Exosomes’ lipidic composition shares similarities to that of membrane lipid rafts, and it includes ceramides, sphingolipids, cholesterol and glycerophospholipids [10,14]. The protein content of exosomes is very broad as it can contain cytosolic or nuclear proteins, transport-involved or adhesion-related and also membrane-bound proteins [10]. Some examples of this wide variety of proteins include heat shock protein (HSP70, HSP90), tetraspanins (CD9, CD63, CD81 and CD82), epithelial cell adhesion molecule (EpCAM) and proteins involved in exosomes biogenesis (ESCRT complex, ALIX, TSG101) [15]. For more specific details on exosomes composition, a manually curated web database containing information about exosomal proteins, RNAs and lipids is available at www.exocarta.org (accessed on 04 October 2021) [16].

### 2.2. Exosomes’ Roles in Metastatic Cancer

Exosomes were initially thought to be a mechanism through which cells could eliminate unnecessary proteins [17,18]. However, the past decades were marked by a notable development in the study of exosomes and it is now demonstrated that these EVs play a critical role in intercellular communication, which is of paramount importance in the context of tumor progression and metastases [8,9,19,20,21].

Even though the mechanisms by which exosomes are taken up by recipient cells is not fully understood, several studies bring evidence of a non-random process which is dependent on transmembrane proteins [22,23]. For example, a study by Kuroda et al. recently identified possible receptors for the uptake of exosomes derived from SK-Mel-28 melanoma cells in human brain capillary endothelial cells (hCMEC/D3) [22]. This study revealed that the uptake of SK-Mel-28-derived exosomes by hCMEC/D3 cells occurs via macropinocytosis and receptor-mediated pathways, with major contribution of the presence of CD46 in hCMEC/D3 [22]. This selective uptake by recipient cells together with the heavily regulated loading process briefly described in the previous section, supports the specific and critical function of these vesicles in intercellular communication.

Recent reports have demonstrated that the successful development of brain metastases rely on a complex intercellular communication occurring between metastatic cancer cells and brain stroma cells, which involves secreted proteins or small vesicles, namely exosomes [24,25]. The existence of various mechanisms by which exosomes are capable of positively or negatively influencing brain colonization by cancer cells is evidence of an immense complex intercellular communication network, which can be considered as a target for new therapeutic approaches or as an inspiration for new drug delivery strategies. In the next sections, we will review some roles of exosomes in tumor progression with focus on intercellular communication within the tumor microenvironment (TME) and in pre-metastatic niche (PMN).

#### 2.2.1. The Tumor Microenvironment (TME): A Dynamic Neighborhood

The TME and its intrinsic complex intercellular communication network, established between stromal and cancer cells, highlights the magnitude of the challenge in understanding and treating cancer. The TME constantly changes during cancer progression as a response to evolving tumors and their oncogenic signals [26]. Therefore, when addressing the formation of metastases, it is necessary to consider the influence of the TME, as its dynamic character allows tumor cells to modulate their own niche. This topic has been reviewed in detail by Quail and Joyce [26].

**Figure 1:**
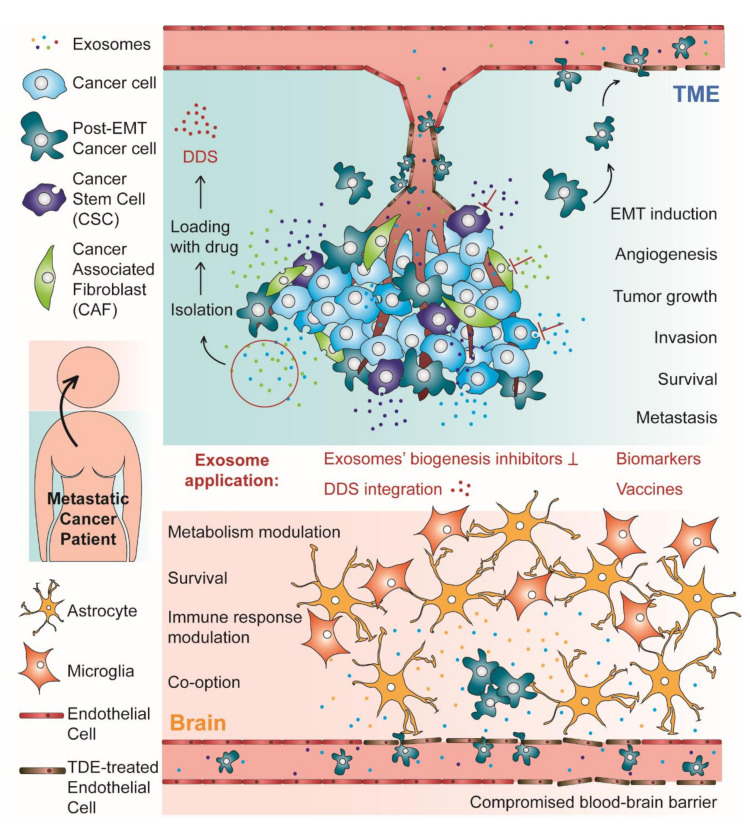
Schematic representation of both stages of the process of metastases formation with tropism to the brain: the tumor microenvironment (TME) and the pre-metastatic niche (PMN)/metastases establishment in the brain. In the TME, tumor derived exosomes (TDEs) are responsible for many critical phenomena, such as epithelial–mesenchymal transition (EMT) and angiogenesis, which supports tumor growth, invasion, survival, and metastases formation. In this stage, the exosomes can also induce endothelial barrier permeation, facilitating the passage of cancer cells to the blood flow. In the brain, exosomes from cancer cells induce many alterations that contribute to PMN establishment, such as blood–brain barrier (BBB) permeation, metabolism and immune response modulation and vascular co-option induction, which supports the brain parenchyma invasion by arriving circulating tumor cells, metastases formation and cancer cells survival. Brain metastases are also supported by the crosstalk between cancer cells and cells from the BBB, such as astrocytes. For their critical role in metastases formation, exosomes can be considered as a promising new target for metastatic cancer therapy, using inhibitors of exosomes’ biogenesis. Alternatively, these extracellular vesicles have been investigated as biomarkers for metastatic cancer diagnosis and prognosis, as vehicles in drug delivery systems (DDS) and as cell-free therapeutic tools in anti-tumor vaccination.

Over the past decades, emerging evidence suggests that tumor-derived exosomes (TDEs) and exosomes derived from stromal cells of the TME are crucial in modulating tumor growth, angiogenesis, invasion, survival, and metastases formation [27,28]. Virtually, TDEs play critical roles in every step of the metastatic cascade. Overall, this process can be considered as having two different stages: the TME stage, where the TDEs induce the epithelial–mesenchymal transition (EMT) in neoplastic epithelial cells conferring them intravasation and migration ability; the PMN stage, which happens in distal and specific organs that will foster metastases [29]. These two stages are represented in Figure 1, which schematically represents the subject reviewed in this work. The role of exosomes in the PMN will be discussed in the next section. In fact, when trying to describe the role of exosomes in brain metastases formation, the most critical and intriguing step may be the transmigration of the BBB and further brain parenchyma colonization by cancer cells. However, before reaching that stage in the metastatic cascade, tumor cells need to first lose their adhesion to the surrounding stroma and enter the bloodstream [29,30].

The metastatic cascade is initiated within the TME, with the activation of EMT process in neoplastic epithelial cells [29]. EMT is the reversible process by which a neoplastic epithelial cell undergoes to acquire mesenchymal features, such as migratory and invasive abilities [29]. During this process, the cells undergoing EMT downregulate epithelial markers, such as cytokeratin and E-cadherin, and upregulate mesenchymal markers as N-cadherin and vimentin [31,32]. Interestingly, the cadherin switch inherent to EMT modulates pro- and anti-apoptotic genes, allowing cancer cells to avoid programmed cell death induced by adhesion loss once they acquire a mesenchymal phenotype [32]. Other relevant features of the mesenchymal phenotype is the increase of matrix metalloproteinases (MMPs) and altered protein production which contributes to the breakdown of the basement membrane [33,34]. Overall, the mesenchymal phenotype renders neoplastic cells the ability to alter their shape and motility, detach from the primary tumor site and enter the bloodstream [35]. Additionally, exosomes secreted during hypoxia, which is linked to EMT and high risk of metastases, are enriched in EMT inducers when compared to those produced in a normoxic state [29].

Recent studies have been building evidence of TDEs involvement in EMT. More specifically, TDEs have been described to transfer considerable amounts of EMT inducers to recipient tumor stroma epithelial cells, which then undergo biochemical changes consistent with EMT [29,36,37,38]. The EMT induction promoted by exosomes can happen via several EMT-related signaling pathways. One of the most studied EMT-inducing signaling pathways is Wnt/β-catenin which is also a common target for exosomes [39,40]. For instance, the transfer of exosomal microRNA (miR)-1260b between lung adenocarcinoma cells leads to downregulation of sFRP1 and Smad4, activating the Wnt/β-catenin pathway [41]. Exosomes derived from cancer associated fibroblasts (CAFs) transferred miR-92a-3p to colorectal cancer cells activating the Wnt/β-catenin pathway and thereby inducing EMT [42]. Furthermore, exosomal miR-92a-3p effect in recipient cells also included apoptosis inhibition and chemotherapy resistance [42], which demonstrates that the crosstalk within the TME dictates tumor progression by modulating several features within the tumor niche. Another line of evidence supporting this TME modulation through exosome-mediated crosstalk is the transfer of miR-155 from breast cancer stem cells (CSCs) and chemoresistant breast cancer cells to sensitive breast cancer cells, leading to marked chemoresistance and inducing EMT [36]. Additionally, a study by Donnaruma and co-workers revealed that several exosomal miRNAs secreted by CAFs were able to induce EMT, facilitate anchorage-independent cell growth and increase the ability to form mammospheres in breast cancer cells [43]. In addition to miRNAs, You and co-workers recently showed that CAFs derived exosomes transferred SNAI1 mRNA to lung cancer cells, inducing EMT via Wnt/β-catenin pathway [44]. Snail1 is a transcription factor which represses the expression of E-cadherin, therefore inducing EMT [44]. In another study, Menck and co-workers described the reciprocal loop between infiltrating macrophages and breast cancer cells. Breast cancer cells secrete exosomes that induce the Wnt ligand Wnt5a in infiltrating macrophages. Macrophages are then responsible to shuttle the Wnt5a to tumor cells, promoting their invasion [45].

Alternatively, exosomes secreted by mesenchymal stem cells (MSCs)-derived adipocytes were able to induce EMT in breast cancer cells via Hippo pathway [46]. Even though in this case the cargo responsible for EMT induction was not identified, other studies showed that activation of the Hippo pathway may result from the transfer of exosomal miRNAs or proteins [47,48,49,50]. Another alternative pathway associated with EMT is the extracellular-regulated protein kinase (ERK) pathway. Exosomes secreted by gastric cancer cells activated the mitogen-activated protein kinase (MAPK)/ERK pathway in recipient cells, leading to tumor proliferation [51].

Collectively, these studies are evidence that the exosome-mediated crosstalk within the TME is crucial for the initiation of metastization process, involving not only TDEs but also exosomes derived from CAFs, macrophages and many other cell types.

#### 2.2.2. Moving Out of the Neighborhood: The Pre-Metastatic Niche (PMN)

The formation of metastases, a not fully understood process, has in the last centuries been explained according to different approaches [52]. In the late 19th century, Stephen Paget proposed the theory of ‘seeds and soil’, where the metastases establishment would result from the interaction between the cancer cells (‘seeds’) and the host microenvironment (‘soil’) [53]. In the early 20th century, James Ewing proposed that the metastatic spread could be explained with the hematogenous flow [54]. Later, in the 1970s Isaiah Fidler’s work brought the two previous theories together and demonstrated that, despite the relevant contribution of the blood flow, the metastatic spread would occur at specific organ sites [55,56]. In fact, Fidler redirected cancer researchers’ attention to the question initially raised by Paget and that remains one of cancer’s greatest mysteries: the mechanisms ruling metastatic organotropism. Since then, a lot of research work intended to solve this conundrum and although progress has been made, several questions remained unanswered [52]. However, an important advance in this field was the demonstration of the existence of PMNs [57]. PMNs refer to pre-established microenvironments occurring in distant organs that favor cancer cells’ settlement and proliferation [9,52]. The discovery of the PMN establishment prior to circulating tumor cells (CTCs) arrival at metastatic sites paved the way for numerous research studies focused not only on the molecular drivers of such process, but also on the molecular and cellular alteration occurring during this process and it is now possible to find several excellent reviews on this matter [26,52,58,59]. Overall, a PMN consists of a series of events, namely vascular leakiness, alteration of local resident cells, recruitment of non-resident cells, extracellular matrix (ECM) remodeling and immune deregulation [52,60]. All these events are induced by tumor-secreted factors and EVs, including exosomes [52,60].

The brain has no classical lymphatic circulation. Therefore, to invade this metastatic site, CTCs must migrate through the BBB to further colonize the brain parenchyma [61]. Therefore, BBB transmigration represents a key event in the process of metastization to the brain [62]. Nonetheless, once cancer cells have transmigrated through the BBB, they reach the brain parenchyma and their progression may also benefit from the complex intercellular communication network occurring at the metastatic niche [63]. In this subsection, we will review some examples of how exosomes may contribute to PMN establishment in the brain in both levels: transmigrating through the BBB and settling in the brain parenchyma. A brief summary of such examples can be found in Table 1.

Several efforts have been made to describe the contribution of exosomes in the process of BBB transmigration by cancer cells [62,67]. One remarkable example of those efforts is the study by Tominaga et al. that showed that EVs, including exosomes, secreted by brain metastatic derivative (BMD) cell populations selected from breast cancer cells MDA-MD-231-luc-D3H2LN transferred miR-181c to brain endothelial cells leading to increased BBB permeability [62]. This BBB breakdown was demonstrated to be a result of the disruption of intercellular junctions promoted by a change in the location of tight junction proteins—Claudin-5, Occludin and ZO-1—N-cadherin and actin filaments [62]. Interestingly, in this study, the expression of miR-181c was analyzed in EVs in sera from breast cancer patients and the results revealed higher miR-181c levels in patients with brain metastases [62]. Using an identical approach, Lu and co-workers demonstrated a similar in vitro effect as that of miR-181c, this time for a long non-coding RNA (lncRNA) [64]. Exosomes derived from breast cancer cells that contained lncRNA GS1-600G8.5, were taken up by brain endothelial cells leading to a reduction of ZO-1, Claudin-5 and N-Cadherin expression [64]. In another study, it was demonstrated that transferring miR-105 via exosomes derived from MDA-MB-231 breast cancer cells to primary human microvascular endothelial cells (HMVECs), resulted in downregulation of ZO-1, leading to tight junction disruption and BBB increased permeability [65]. Another line of evidence of exosomes contributing to BBB breaching was recently brought by Kinjyo and co-workers as they showed that precursor B acute lymphoblastic leukemia (BCP-ALL) blasts release exosomes to the blood, and cultured endothelial monolayers treated with exosomes derived from BCP-ALL cells allowed transmigration of leukemia cells [67]. Moreover, cultures of astrocytes treated with BCP-ALL cells derived exosomes resulted in increased production of vascular endothelial growth factor-A (VEGF-AA), which has been reported to be associated with leukemia infiltration into the central nervous system (CNS) [67]. All these studies support the role of exosomes in facilitating the BBB transmigration by cancer cells, through compromising BBB integrity prior to CTCs arrival.

After BBB transmigration, cancer cells’ survival and progression may be supported by the complex intercellular communication network occurring at the brain parenchyma [63]. Fong and co-workers demonstrated that breast cancer cells specifically secrete miR-122 into EVs, including exosomes [68]. In this study, it was shown that primary astrocytes efficiently uptake EVs derived from breast cancer cells with high levels of miR-122, leading to an increase in miR-122 intracellular levels, due to direct transfer and not by induction of its expression [68]. This leads to a decrease in PKM2 (pyruvate kinase) and GLUT1 (glucose transporter) expression, and consequent reduction in PKM activity and 2-NBDG (a glucose equivalent) uptake [68]. Interestingly, these effects were confirmed in vivo by intravenous injection of breast cancer cells derived vesicles containing exosomes in mice, followed by glucose uptake measurement in brain and lungs. In both sites, there was a decrease in glucose uptake and reduced expression of PKM and GLUT1 [68]. Additionally, an intracardiac injection of luciferase-labeled MDA-MB-231-HM cells in mice pretreated with vesicles derived from breast cancer cells resulted in significant metastatic colonization in lungs and brain after three weeks [68]. This work clearly shows the specific role of vesicular miR-122 in modulating the glucose metabolism of resident cells within the PMN, which promotes metastatic colonization by increasing the levels of glucose available to incoming CTCs [68]. As it is known, the Warburg effect in cancer cells results from the preferential aerobic glycolysis in glucose metabolism even when the levels of oxygen are sufficient [68]. This effect translates in an increased glucose uptake by cancer cells and hence, high levels of available glucose confer these cells an advantage within the PMN.

The ability of CTCs to establish contact with and spread along brain endothelial cells, a process named vascular co-option, is also described to be relevant in the process of metastization [69,73]. A recent study by Rodrigues and co-workers unraveled the role of exosomal cell migration-inducing and hyaluronan-binding protein (CEMIP) in brain metastization by breast cancer cells [69]. Exosomes derived from brain tropic breast cancer cells are enriched with CEMIP, which promotes vascular co-option, and therefore, successful invasion and metastatic colonization of the brain [69]. Moreover, it was demonstrated that loss of CEMIP hinders the interaction between brain metastatic cells and brain vasculature and consequently brain invasion [69]. Additionally, in vivo experiments also revealed that exosomal CEMIP is involved in molecular pathways of brain endothelial cells and microglia that are associated with brain metastases establishment [69]. Interestingly, data obtained from quantification of CEMIP in patient samples showed that metastatic tumors with high levels of CEMIP are linked to poor survival when compared to those with low levels of CEMIP [69].

In another study, it is shown that X-inactive–specific transcript (XIST) knockdown in breast cancer cells led to exosomal secretion of miR-503 [70]. MiR-503 promotes M1-M2 conversion of microglia and increases its programmed cell death ligand 1 (PD-L1) expression, inducing local immunity suppression, which enhances tumor growth [70]. In this case, exosomes contribute to brain metastases progression by modulating the immune response within the PMN. Another TDEs’ contribution to the PMN formation is recruitment of bone marrow derived cells (BMDCs), since these cells have the ability to modulate homing of primary tumor cells to metastatic sites through the crosstalk between these two cell types [71]. Peinado and co-workers demonstrated that melanoma cells transfer the MET oncoprotein to bone marrow progenitor cells via TDEs, supporting the metastatic cascade in vivo [71].

The intercellular communication between cancer cells and cells from the metastatic niches is a reciprocal process. In a recent work by Xu and co-workers, it was shown that exosomes derived from human brain microvascular endothelial cells (HBMECs) were uptaken by small cell lung cancer (SCLC) cells [66]. As a result, SCLC recipient cells were capable of evading death signals under oxidative stress, increasing the cell survival rate which would facilitate the colonization of the brain parenchyma [66]. In this work, S100A16 is pointed out as one of the molecules responsible for this effect since its levels were increased in SCLC cells upon HBMEC-derived exosomes uptake [66]. However, the increase in S100A16 levels in SCLC recipient cells was shown to be a result of its induced overexpression and not a direct transfer from HBMEC-derived exosomes [66]. Interestingly, S100A16 overexpression in MCF-7 breast cancer cells is also linked with EMT via Notch-1 pathway [74]. Furthermore, S100A16 coexpression with S100A14 is associated with poor prognosis of breast cancer patients and invasive activity of breast cancer cells, promoted by an interaction with the cytoskeleton dynamics [66]. Finally, Zhang and co-workers demonstrated that astrocytes derived exosomes contained high level of miR-19a and are uptaken by brain metastatic breast cancer cells (MDA-MB-231Br), leading to a downregulation of PTEN mRNA [72]. PTEN loss in brain metastases leads to increased chemokine CCL2 expression, which results in myeloid cell recruitment [72].

## 3. Exosomes and Metastatic Cancer Treatment

Considering the relationship between exosomes and marked severity/aggressiveness of many different types of cancer, one therapeutic approach relies on controlling exosome circulation within the system. Alternatively, exosomes can be considered as an opportunity to develop a new therapeutic strategy that consists of their use as vehicles for anticancer drugs, taking advantage of their natural features that make them natural nano-sized carriers. Therefore, as pivotal participants in intercellular communication, and in the context of cancer treatment, exosomes can be considered a target or a vehicle. In this section we will first overview some studies where exosomes were used as a target, and then focus on how these EVs can be used as DDS and other alternative therapeutic strategies and applications.

### 3.1. Targeting Exosomes as a Therapeutic Strategy

When aiming for modulation of the exosome circulation, one obvious step to consider is inhibiting exosomes’ biogenesis. Due to exosomes’ biogenesis complexity, it is challenging to develop a molecule that effectively blocks this process [75]. Moreover, besides being associated to the development of pathological conditions, exosomes and EVs are also participants in physiological processes, such as stem cell maintenance, tissue repair, immune surveillance, and blood coagulation [76,77]. Therefore, when considering targeting exosomes by inhibiting their biogenesis, the challenge resides in identifying and effectively blocking specific subpopulations linked to a particular disease, avoiding the impairment of biological functions played by these EVs.

The use of inhibitors of formation and release of EVs, including exosomes, is well revised elsewhere [75]. Overall, the inhibition of the exosomes’ biogenesis can generally occur by inhibiting trafficking or release of vesicles [75]. One example of an EVs trafficking inhibitor is manumycin A, a farnesyltransferase inhibitor which is also a cell-permeable antibiotic extracted from *Streptomyces parvulus* [75]. Manumycin A inhibits Ras farnesyltransferases. Ras refers to a family of small GTPases which is involved in many cellular processes, including exosomes release, and its de-regulation in cancer cells correlates with increased invasiveness, metastases formation and reduced apoptosis [75]. Therefore, manumycin A has been used as an inhibitor of exosomes secretion [78,79,80]. Other examples of inhibitors of EVs trafficking are calpeptin and Y27632 [75]. Regarding the inhibition of exosomes’ biogenesis by blocking their release, the most widely used inhibitor is GW4869 [81,82,83,84,85,86,87]. This molecule is a potent non-competitive inhibitor of membrane neutral sphingomyelinase (nSMase). nSMase generates bioactive ceramide via hydrolysis of sphingomyelin [75,83]. Ceramide is one of the most common lipids in the exosomes lipid bilayers and it plays an important role in ESCRT-independent exosome generation [75]. Therefore, GW4869 has been reported to inhibit exosome release [75]. However, since SMase is found in several cellular compartments, as the Golgi apparatus, endosomes and cell membrane, its activity is also related to microvesicles (MVs) shedding [75]. Interestingly, a work performed by Menck and co-workers demonstrated that exosome inhibition and MVs release are linked [87]. In this work, breast cancer cells SKBR-3 were treated with GW4869 or siRNAs against sphingomyelin phosphodiesterase 2/3 (SMPD2/3) [87]. Overexpression of SMPD2 or SMPD3 led to a decrease in larger vesicles and an increase in smaller vesicles release [87]. Treatment with GW4869 or siRNAs led to an increase in vesicles with sizes ranging from 100 to 200 nm and a decrease in vesicles with smaller sizes [87]. Another example of an inhibitor of EVs release is imipramine, a tricyclic anti-depressant with the ability to inhibit acid sphingomyelinase (aSMase), therefore impairing exosomes’ release and MVs generation [75].

Inhibiting exosome secretion can also be accomplished by silencing pivotal players in this process. For example, Bobrie et al. knocked down Rab27a/b in two mammary carcinoma cell lines—4T1 (metastatic) and TS/A (nonmetastatic)—and showed that, in vivo, both primary tumor growth and lung dissemination of 4T1 were significantly decreased [88]. More examples of GTPases that may represent alternative targets for inhibiting exosome release by interfering with MVB docking or fusion with the cytoplasmic membrane are Rab11 and Rab35 [75,76]. Other less common molecules have also been investigated for their ability to inhibit EVs release acting in different targets within the multiple cascades involved in this complex biogenic process [75,76].

Another theoretical approach to target exosomes and interfere in cancer progression and metastatic cascade is to prevent exosomes’ uptake by the recipient cells. One major limitation to this approach is the fact that the precise mechanism for EV trafficking and target definition remains to be unraveled and, more importantly, several reports point to different uptake mechanism depending on the exosomes and the recipient cells [76,89]. However, some EVs uptake inhibitors can be found in the literature. One of those inhibitors is diannexin, which interferes with EVs uptake by blocking surface phosphatidylserine (PS) which is relevant for cell adhesion [90,91]. Nonetheless, it is important to mention that PS exposure at the membrane is not an exclusive feature of EVs and it is also found in apoptotic cells and activated/angiogenic endothelium [76,91]. Therefore, this lack of specificity poses an obstacle to the widespread application of this inhibitor. Another inhibitor of exosomes’ uptake is heparin, which was demonstrated to interact with cell-surface heparan sulfate proteoglycans (HSPGs), therefore competing with exosome binding in these receptors [92]. In another study, heparin efficiently blocked the transfer of brain tumor cells derived EVs into recipient cells by interfering with EVs uptake in more than one way [93]. Heparin was found to be co-localized with EVs in microscopy and EVs aggregation in the presence of heparin was also observed in transmission electron microscopy (TEM) [93]. Interestingly, in vivo studies have reported a decrease in metastases after injection of heparin. The mechanism underlying the antimetastatic ability of heparin is thought to be the blockade of the interaction between tumor cells and platelets, which is deemed important within the metastatic cascade [93,94,95].

A more extreme approach is the removal of exosomes as a therapeutic adjuvant through an extracorporeal hemofiltration method. The biotechnology company Aethlon Medical (San Diego, CA, USA) has developed a hemofiltration system called ADAPT^TM^ (Adaptive Dialysis-like Affinity Platform Technology). This approach consists of hollow-fiber plasma separator cartridges with immobilized affinity agents that interact with target molecules in the exosomes surface and selectively adsorbs them, while blood cells and non-bound serum components flow through the device [96]. Although this strategy would not offer drug toxicity, which is an advantage when compared to the other pharmacological approaches, it requires patients to undergo surgical procedures for vascular access, which is not ideal [96].

### 3.2. Exosome Isolation

Several methods are available for exosome isolation, from cells or body fluids, focusing on different features of these vesicles, such as size, shape, density, solubility, or surface markers [97,98,99,100]. Generally, an exosome isolation from cell culture process initiates by culturing the producer cells with exosome-free media (usually serum-free media or complete media with exosome-depleted fetal bovine serum (FBS)), allowing them to condition the media for a determined period of time [97,99]. The conditioned media is then collected and processed according to the protocol that better suits the isolation technique to be used [97]. Regarding exosome isolation from body fluids, the methods available are those applied in isolation from cell culture. Occasionally, fluid sample dilution and pre-clean may be necessary to decrease viscosity and remove discardable large particles, respectively [97,99]. Protease inhibitors can be used to prevent exosomal protein degradation [97].

Ultracentrifugation (UC) is the most widely used technique and it can be performed as one of the two types: differential or by density-gradient [97,99,100]. It is important to mention that, as UC is mainly based on size, and considering that the size of exosomes overlaps with MVs’ size, the purity of the exosome sample obtained may be questionable [97]. This is an inherent limitation of the use of exosomes that will be further discussed in another section of this review.

Additionally based on size, ultrafiltration can be used to isolate exosomes by sequential filtration through membrane filters with specific size-exclusion thresholds [97,98,100]. Another size-based isolation technique is the size-exclusion chromatography [97,100]. In this technique, a column is packed with beads with pores smaller than exosomes and these are segregated from smaller and bigger particles [97,100]. These size-based isolation techniques are less time consuming than UC, but they also face the limitation of size-overlap between exosomes and other MVs [97].

Another exosome isolation technique is immunoaffinity capture-based which relies on the use of antibodies for specific exosomes surface proteins [98,100]. The immunoaffinity procedure consists of the immobilization of the antibodies for selected exosomes’ surface markers in a support media, such as magnetic beads, chromatography matrices, ELISA plates or microfluidic devices [97,99,100,101]. Although this method requires much less sample volume, the yield is comparable to that of exosome isolation by UC [97].

Exosome isolation by precipitation consists of the use of polymers that can alter exosomes’ solubility, resulting in their precipitation [97,98,100]. More specifically, polymers bind to water molecules and promote the precipitation of the less soluble exosomes, which can be collected by low-speed centrifugation [97,98,100].

The common challenge to all these isolation methods is sample purification. Efficient separation of exosomes from all the other EVs, protein aggregates or cellular debris is not trivial. Therefore, it is deemed important that each exosome batch is characterized before further application. Exosome characterization is performed with focus on several characteristics, such as exosome size, size distribution, protein concentration, specific surface markers and morphology [97]. Another general limitation is the efficiency of the exosome isolation process, since for most of these techniques results in low production yield. Although polymeric precipitation may result in higher production yields, the purity of the sample obtained with this technique is compromised since it does not separate the exosomes from the polymeric reagent used [97,102,103].

### 3.3. Drug Loading

The currently available drug loading methodologies for exosome and MVs in general can be divided into two main groups: pre-isolation and post-isolation. In pre-isolation methodologies, the cargo is either produced by or loaded into the producer cells and hence, the isolated MVs will be pre-loaded in advance [97]. On the other hand, in post-isolation methodologies the MVs are firstly isolated from the producer cells and then the drug is loaded into the vesicles (exosomes or MVs) [97].

Regarding drug loading by pre-isolation methodologies, it can happen by simply treating the producer cells with the drug intended to load and the cells will naturally secrete drug pre-loaded MVs [97]. In this case, it is impossible to control the loading efficiency, yet this method is widely used due to its simplicity [97]. Another approach to load drugs into MVs through pre-isolation methodologies is producer cells’ engineering, mostly done by transfection or activation of these cells [97]. Engineering cells by transfection is the most commonly used and efficient method to load oligonucleotides into exosomes. Interestingly, RNAs and protein sequences can be easily transfected as oligonucleotides or a plasmid backbone [97]. Moreover, transfection can also be used to induce overexpression of a specific protein in the surface of the exosomes [97]. Alternatively, drug loading by cell activation is not a very effective method, but it has been shedding some light on the physiology and function of exosomes [97].

Several methods can be used to load drugs into exosomes in a post-isolation manner. The simplest method consists of the direct incubation of exosomes with the drug. The main disadvantage of this method is the low loading efficiency, that will depend on the lipophilic properties of the drug and the concentration gradient [97,104,105]. Another drug-loading technique is electroporation. Here, the phospholipid bilayer of the MVs is disturbed by applying an electrical field which creates small pores, allowing the passage of the drug into the vesicles [97]. This technique is often used to load siRNAs into exosomes [97,106,107]. Notably, the success of the drug-loading through electroporation depends on the recovery of the membrane integrity of the vesicles. In a study by Kooijmans et al., it was demonstrated that electroporation resulted in exosome membrane disruption and considerable siRNA aggregation which will lead to overestimation of the loading efficiency [108]. Nonetheless, efforts have been made to surpass this technical issue. Johnsen and co-workers loaded exosomes using electroporation and an optimized buffer, reporting that exosomes’ structural integrity was not compromised, and aggregation was prevented [109]. In addition, sonication can also be used to load drugs into exosomes. In this technique, a probe sonicator is applied to the mixture of the exosomes and the drug, and induces membrane deformations, resulting in drug incorporation into the vesicles [97,110]. Alternatively, the mixture of exosomes and the drug can be subjected to extrusion through membranes with porous sizes from 100 to 400 nm [97]. During extrusion, drug loading into exosomes happens as a consequence of exosomes’ membrane disruption in the presence of the drug [97]. Fuhrmann and co-workers loaded exosomes with porphyrins using extrusion and reported that porphyrin-loaded exosomes exhibited a significantly altered zeta potential compared with original exosomes, probably due to critical alterations in the membrane [111]. Interestingly, porphyrin-loaded exosomes obtained using extrusion exhibited significant cytotoxic activity, while porphyrin-loaded exosomes obtained with other techniques did not [111]. Freeze/thaw cycles can also be performed to promote drug loading into exosomes [97,112]. Nonetheless, the loading efficiency obtained with this method is usually lower when compared with extrusion or sonication [97]. Saponin-assisted loading is a method that relies on chemical induced permeation of exosome membranes [97]. A major drawback of this method is the hemolytic activity of saponin. Even though the precise mechanism by which saponin is able to damage red blood cells is not described yet, the saponin concentration needs to be kept as low as possible and it should be removed upon incubation with exosomes [97,113].

### 3.4. Using Engineered Exosomes as DDS

As an alternative to target exosomes, these EVs can be used as vehicles in DDS. In fact, much evidence is rising to support the promising application of exosomes in drug delivery. This promising role is also strongly reinforced by specific features of exosomes which were previously discussed in this review and extensively reviewed elsewhere, which include small size, nontoxicity, long circulation, low immunogenicity and ability to cross biological barriers as the BBB [7,9,76,97,114]. Although the precise mechanism by which exosomes transmigrate through the BBB is not yet generally described, several studies have been trying to answer this question, demonstrating that BBB transmigration by exosomes is mainly happening via transcytosis [115,116]. In a work performed by Morad et al., exosomes were isolated from a brain-seeking variant of MDA-MB-231 breast cancer cells (Br-EVs) and their transmigration route was investigated in vitro and in vivo [115]. Morad and coworkers used an in vitro static BBB model, a microfluidic organ-on-a-chip model of the BBB (BBB chip) and zebrafish embryos and demonstrated that Br-EVs transmigrated the BBB via transcytosis, without compromising its integrity, through a caveolin-independent mechanism, involving recycling endosomes and basolateral SNAREs [115]. Some recent reports have demonstrated exosomes’ potential application in cancer treatment with promising results [105,106,117,118]. In this section, we will outline some of those reports, keeping focused in works with major direct/indirect impact on brain metastases.

In a pioneering study by Alvarez-Erviti et al., exosomes were isolated from dendritic cells (DCs). These exosomes were engineered with peptides from muscle—a muscle-specific peptide (MSP)—and brain tissue–CNS-specific rabies viral glycoprotein (RVG), to favor targeting of these tissues and allow gene therapy, to prevent degenerative diseases [106]. MSP exosomes and RVG exosomes were loaded with nonspecific Cy5-labeled Glyceraldehyde-3-phosphate dehydrogenase (*GAPDH*) siRNA using electroporation [106]. Murine muscle (C2C12) and neuronal (Neuro2A) cells were treated with Cy5-labeled *GAPDH* siRNA alone (siRNA), or siRNA and Lipofectamine 2000 (siRNA + LP), or unmodified exosomes with siRNA or MSP/RVG exosomes loaded with siRNA [106]. The results clearly showed that the gene knockdown was comparable in cells treated with MSP/RVG exosomes loaded with siRNA and siRNA + LP, meaning that exosome-mediated delivery of the siRNA was as efficient as state-of-the-art transfection reagents [106]. Importantly, the knockdown was cell-specific, since the strongest effect of siRNA delivery by MSP exosomes was obtained in C2C12 cells, and by RVG exosomes in Neuro2A cells [106]. The hypothesis of using these exosomes to systemically deliver siRNA in vivo was further evaluated. Delivery of siRNA led to knockdown in the spleen, liver and kidney, consistent with typical siRNA sequestration resulting from tail vein delivery [106,119]. On the other hand, siRNA-loaded exosomes showed no significant effect in these organs, since non-specific uptake was not significant [106]. Notably, injection of RVG exosomes resulted in significant knockdown of GAPDH mRNA in several brain regions [106]. This work is a remarkable example of the exosomes’ ability to deliver cargo across the BBB and how engineered exosomes constitute a promising vehicle for specific drug delivery.

Recent studies have been using exosomes as DDS for glioblastoma treatment [117,120,121,122,123]. Yang and co-workers isolated exosomes from brain neuronal glioblastoma-astrocytoma U-87 MG, endothelial bEND.3, neuroectodermal tumor PFSK-1 and glioblastoma A-172 cell lines [117]. In this study, exosomes were loaded with rhodamine 123, paclitaxel or doxorubicin through direct incubation [117]. Cytotoxic effects of paclitaxel and doxorubicin delivered by bEND.3- or U-87 MG- derived exosomes were evaluated in U-87 MG cell cultures [117]. While paclitaxel, doxorubicin or exosomes alone did not cause a significant cytotoxic effect, paclitaxel and doxorubicin delivered through exosomes showed a significant cytotoxic effect in U-87 MG cells [117]. The ability of drug delivery across the BBB using these exosomes was studied by following the delivery of rhodamine 123 via exosomes or alone, in a primary brain cancer model obtained in zebrafish embryos by injecting U-87 MG cells into the brain ventricle [117]. After 18 h of injection via the cardinal vein, rhodamine 123 alone remained in the vessels and it was not observed in brain tissue [117]. The same result was observed for rhodamine 123 delivered through U-87 MG-, PFSK-1- and A-172-derived exosomes [117]. However, rhodamine 123-loaded bEND.3-derived exosomes were able to cross the BBB and enter the brain [117]. Therefore, the authors studied the delivery of the anticancer drugs into the brain via bEND.3-derived exosomes in vivo [117]. Zebrafish embryos treated with doxorubin-loaded bEND.3-derived exosomes showed a significantly smaller area with U-87 MG cancer cells and very few cancer cells in the brain [117]. Moreover, exosomes loaded with doxorubicin also led to vascular endothelial growth factor (VEGF) RNAs suppression in the brain [117]. This study clearly shows that bEND.3-derived exosomes have a significant therapeutic efficacy in delivering doxorubicin into the brain and across the BBB in a zebrafish brain cancer model [117]. In a similar work, Yang and co-workers obtained bEND.3 cells derived exosomes and loaded them with VEGF siRNA using Lipofectamine^®^ 2000 transfection reagent [122]. By using an in vitro model—Transwell^®^ filters with astrocytes in the abluminal side and bEND.3 on the luminal side—and an in vivo model—xenotransplanted cancer cells in zebrafish—the authors demonstrated the ability of exosomes to deliver its cargo across BBB and effectively inhibit tumor growth [122]. Erkan et al. loaded HEK293T cells derived EVs with cytosine deaminase (CD) and uracil phosphoribosyltransferase (UPRT) suicide gene products. In this work, the approach was the delivery of CD-UPRT, as the machinery responsible for converting 5-fluorocytosine (5-FC) into 5-fluorouracil (5-FU), which then leads to defective DNA replication and apoptosis [120]. This strategy led to significant results in an in vivo model obtained from subcutaneous xenograft of glioblastoma in mice, leading to approximately 70% reduction in tumor growth [120]. One key difference between these studies relies on the administration method used to introduce the DDS in vivo. While in the studies by Yang et al. the administration was performed via the cardinal vein, Erkan et al. used a direct intratumoral injection, which does not allow to draw conclusions regarding the route for EVs delivery and may be impractical in non-accessible tumors [117,120,122].

In a recent study from Liu et al., exosomes were isolated from MSCs transduced with lentiviral chemokine receptor CXCR4 and lentiviral tumor necrosis factor-related apoptosis-inducing ligand (TRAIL) (Exo^CXCR4 + TRAIL^) [124]. CXCR4 and its ligand SDF-1 are commonly expressed in immune, brain and heart cells [125]. Moreover, SDF-1/CXCR4 are involved in stem cell homing, engraftment, expression of adhesion molecules, chemotaxis, proliferation, and survival [124,126,127]. TRAIL is a member of the tumor necrosis factor ligand family, and it is linked with apoptosis activation. Although TRAIL is expressed on the cell surface, it can be released as soluble TRAIL, which can cause apoptosis in tumor cells, but not in cells from healthy tissues [128]. In this study, the anti-tumor effect of carboplatin injected in combination with Exo^CXCR4 + TRAIL^ (carboplatin + Exo^CXCR4 + TRAIL^ group) or exosomes (carboplatin + exosomes group) was evaluated in breast cancer brain metastases, in vivo [124]. The results revealed a decrease in tumor volume in mice treated with carboplatin +Exo^CXCR4 + TRAIL^, suggesting that the presence of Exo^CXCR4 + TRAIL^ enhances the anti-tumor effect of carboplatin [124]. In this case, there is evidence that engineered exosomes may improve the efficacy of chemotherapy, shedding light on a new approach that relies on the use of synergistic protocols with anticancer drugs to treat brain metastases.

Melzer and co-workers recently loaded MSCs-derived exosomes with taxol, to target metastatic breast cancer and other carcinoma cells [118]. In this work, exosomes were loaded via pre-isolation, by incubating the producer cells with the maximum acceptable taxol concentration [118]. Taxol-loaded MSCs-derived exosomes were applied to breast, ovarian and lung cancer cell cultures resulting in a drastic decrease in cell viability by comparison with untreated cells or cells treated with control MSCs-derived exosomes [118]. An in vivo breast cancer model was obtained using aggressively metastasizing MDA-hyb1 breast cancer cells to induce tumors in NODscid mice [118]. Mice were then treated with taxol-loaded exosomes, leading to a reduction of the average tumor weight in 64% [118]. Even though no metastases were found in the brain, mice treated with taxol-loaded exosomes displayed half of the metastases in comparison with control mice [118]. Therefore, taxol-loaded MSCs-derived exosomes promoted the inhibition of primary tumor growth and consequent metastases [118].

In another study, exosomes isolated from EL-4 cells were loaded with signal transducer and activator of transcription 3 (Stat3) inhibitor JSI-124 (Exo-JSI124) by direct incubation and intranasally delivered to mice bearing intracerebral tumors [105]. Exo-JSI124 treated mice showed less invasiveness and significantly increased survival, since 2 out of 10 survived until day 90—when they were killed—without any neurological symptoms and any evidence of tumor at the original implantation site [105]. Moreover, the results from this study suggest that, mechanistically, exo-JSI124 is selectively taken up by microglia, inhibiting the expression of inflammatory cytokines IL-1β and IL-6 [105].

### 3.5. Exosomes as an Inspiration for New Therapeutic Strategies

#### 3.5.1. Messages in a Bottle: Exosomes as Biomarkers

Exosomes may also be considered as biomarkers in diagnosis and prognosis. These EVs are widely secreted by cancer cells and they can be found in body fluids, such as blood, saliva and urine [43,129]. The potential of exosomes to report on brain cancer was demonstrated by Skog et al., in 2008. In this study, MVs were isolated from cerebrospinal fluid (CSF) of glioblastoma patients and revealed the presence of mutant EGFRvIII RNA [130]. Interestingly, EGFRvIII RNA mutations were identified in MVs from the CSF of two patients with tissue analysis negative for such mutation [130]. Other studies have been correlating the presence of specific proteins and mRNAs or miRNAs in EVs with glioblastoma patients’ prognosis [131,132]. For example, EVs with annexin V found in glioblastoma patients treated with chemoradiation were shown to correlate with earlier recurrence of the disease [132]. Emerging novel techniques, such as single EV analysis and microfluidics, have been improving EVs detection for glioblastoma diagnosis with promising results [133,134,135].

A recent systematic review of the clinical significance of exosomes as potential biomarkers in cancer included 47 diagnostic and 50 prognostic markers from 30 and 42 studies, respectively [129]. Among these, and in the case of the diagnostic markers, 42.6% were miRNAs, 36.2% were lncRNAs and 19.1% were proteins [129]. Similarly, for prognostic markers, 60% were miRNAs, 18% were lncRNAs and 16% were proteins. Regarding sample origin, these exosomal diagnostic or prognostic markers were found in many body fluids, such as serum, plasma, urine, saliva and bile [129]. The meta-analysis results suggest that diagnostic markers allowed to effectively discriminate between cancer patients, non-cancer patients and healthy people [129]. Concerning the prognostic markers, the primary endpoints were defined as overall survival (OS), disease-free survival (DFS) and recurrence-free survival (RFS) in 92.9, 26.2 and 9.5% of the studies [129]. The results from the meta-analysis revealed that exosomes were associated with all the three parameters (OS, DFS and RFS) in various types of cancer [129]. Nevertheless, this meta-analysis excluded studies with more than one biomarker and tissue-based biomarkers [129].

Recently, exosomes from melanoma patients’ T cells and DCs with increased levels of the immune checkpoints Programmed Cell Death Protein 1 (PD-1) and CD28 were found to be correlated with improved treatment response [136]. Determination of S100B and Melanoma Inhibitory Activity (MIA) levels in exosomes from melanoma patients may also be used as an alternative to their analysis in serum for both diagnosis and prognosis purposes [137]. The presence of higher levels of leucine-rich a-2-glycoprotein (LRG1) in exosomes collected from the urine of non-small cell lung cancer (NSCLC) patients correlated with its high expression in tumor tissue, suggesting LRG1 may be a candidate marker for NSCLC in urinary exosomes [138]. In a recent study, several proteins were identified in lung cancer patients’ saliva as potential markers for lung cancer detection [139]. Many other proteins and nucleic acids have been identified as potential markers for diagnosis, prognosis, monitoring or recurrence for multiple types of cancers [140]. In breast cancer, more specifically, it is possible to identify several proteins and nucleic acids with demonstrated clinical relevance as biomarkers for diagnosis, prognosis, and recurrence [140]. All these studies support the use of exosomes in cancer detection and prognosis biomarkers, which will undoubtedly contribute to metastatic cancer treatment by allowing early detection and accurate monitoring of disease progression.

#### 3.5.2. Exosomes as Vaccines

An alternative approach to use exosomes in brain metastases or brain cancer is the use of vaccines. For example, Bu et al. showed that DCs treated with EVs derived from glioma cells were able to activate anti-tumor response from T cells, both in vitro and in vivo [141]. This approach has been widely explored with several types of cancers and immune cells, and it was recently very well reviewed by Naseri et al. [142]. Amongst all the possible strategies for anti-tumor vaccination, the use of DCs is one of the most promising [142]. In terms of personalized tumor immunotherapy, TDEs have been emerging as promising cell-free therapeutic tools for their tumor antigens content. Moreover, TDEs can be systemically collected, their membrane favors cell interaction and attachment and they efficiently deliver their cargo, inclusively across the BBB [7,76,142]. The efficacy of DCs treated with TDEs to induce immune cells responses in vitro and in vivo has been demonstrated with cytotoxic T lymphocytes (CTLs) and T helper cells, B cells, natural killer (NK) cells and macrophages [142]. Nonetheless, establishment of DCs requires safety conditions that are both expensive and time consuming, which impairs this promising clinical application [142]. In the same way, more studies to assure efficacy and minor side effects associated with the use of TDEs would also contribute to advances in this area [142].

## 4. Limitations and Future Perspectives

One of the biggest limitations when working with exosomes resides on accurately identifying the vesicle which one is working with. Notably, great efforts have been made to describe and classify many types of vesicles and two databases are now available: Vesiclepedia (http://microvesicles.org/-accessed on 04 October 2021) and Exocarta (http://www.exocarta.org/-accessed on 04 October 2021). However, technical limitations in samples’ purification often leads some authors to use the term ‘microvesicles’ to refer to a vesicle population which includes MVs and exosomes (excluding apoptotic bodies). Therefore, the use of the term ‘microvesicles’ and ‘exosomes’ is not consistent in the literature. In this review, we generally opted to use the same term as in the mentioned published work.

Regarding technical limitations and with respect to exosome isolation, most of the techniques render a very poor production yield, which poses an obstacle for exosome research work and application in the clinics [97,102,103]. Limitations of the use of exosomes in liquid biopsies, for instance, are mostly related with the need to establish an optimal isolation method [9,129]. UC is the standard method for exosome isolation and despite the fact that its application to tissues is very simple, for body fluids additional steps are required to clean the samples, which is a challenging task [9,97,129]. Other isolation methods have poor robustness, such as sucrose gradients, size exclusion chromatography and microfluidic devices, or lack specificity, such as affinity-based exosome isolation kits [9]. A recent technique, asymmetric flow-field-flow fractionation (AF4) provides rapid results with good reproducibility, but its complexity requires technical expertise when operating the equipment and analyzing the data [9,143]. Therefore, for exosomes to become a recurrent tool in diagnosis and prognosis assessment in the clinics, new or improved isolation methods that are robust, reproducible, specific, and easily available for general use are imperative [9,129]. Another major contribution for the advances in exosome application in the clinics would be the establishment of robust and scalable manufacturing processes. In this matter, a recent work was focused on optimizing MSC EVs production from dynamic cell cultures using different scalable platforms [144].

Despite the notable expansion in the fields of exosomes’ biology and application in biotechnology, there are still a considerable number of challenges to be tackled. Mechanisms of cell uptake, for example, remain not clearly described and they are crucial in the context of drug delivery. In most of the works where exosomes were used as DDS and effectively deliver its cargo across the BBB, the routes and mechanisms underlying BBB crossing are not described [106,117]. Additional efforts in this area would be greatly appreciated, since the complexity of EVs uptake and trafficking requires dedicated works, which are hardly integrated in the studies where the application of exosomes as DDS is investigated. In fact, the few clinical studies focused on the application exosomes in metastatic brain tumors’ diagnosis and therapeutic approaches is a result of the limited number of mechanistic studies with focus on the role these EVs in the TME, cellular uptake by recipient cells and BBB crossing. Progress in understanding the mechanisms behind such role and trafficking routes would strongly impact the development of effective exosomal-based DDS for brain metastases.

## 5. Conclusions

Transfer of tumor factors via exosomes supports both primary tumor growth and metastases formation and this critical role renders these EVs diagnostic and prognostic value, also offering a plethora of new therapeutic options for metastatic cancer, including brain metastases. However, many factors, such as the production/isolation yield, loading efficiency, selective targeting, lack of standardized protocols of isolation/characterization and effective methods for production scale-up remain to be improved. Moreover, several mechanistic details of the exosomes’ biological and pathological functions remain unknown. Nevertheless, the field of exosomes and EVs in general has been remarkably evolving considering its short age and the new insights brought to light by this field strongly impacted in the recognition of the importance of intercellular communication in cancer development, paving the way for new therapeutic approaches.

## Figures and Tables

**Table 1:** Summary of some examples of the role of exosomes in PMN establishment in the brain. The intercellular communication mediated by exosomes is reciprocal: exosomes can transfer molecules from cancer cells to brain resident cells, or vice versa and it can facilitate PMN establishment at the level of BBB transmigration or brain parenchyma colonization.

Location	Exosomes’ Origin	Transferred Molecule	Recipient Cells	Effect	Reference
BBB	MDA-MB-231-luc-D3H2LN (breast cancer cells) and brain metastatic derivative cell lines	miR-181c	Primary brain capillary endothelial cells	BBB breakdown: changed the localization of tight junction proteins, N-cadherin and actin filament	[62]
	MDA-MB-231-luc-D3H2LN and brain metastatic derivative cell lines	lncRNA GS1-600G8.5	BMECs (Human Brain Microvascular Endothelium Cells)	BBB breakdown: decreased expression of ZO-1, claudin-5, and N-cadherin	[64]
	MDA-MB-231MCF 10A	miR-105	HMVECs (Primary Human Microvascular Endothelial Cells)	BBB breakdown: decreased expression of ZO-1	[65]
	HBMEC	S1000A16	SCLC (Small Cell Lung Cancer)	Preservation of mitochondrial membrane potential (ΔΨm) supporting SCLC survival in brain	[66]
BBB + Brain parenchyma	NALM6 (Precursor B Acute Lymphoblastic Leukemia (BCP-ALL) cell line)	IL-15	bEnd.3 (mouse brain endothelial cells) and astrocytes	Disruption of BBB integrity + alteration of the activation state of resident cells in the brain	[67]
Brain parenchyma	MDA-MB-231 MCF 10A	miR-122	Astrocytes	Glucose metabolism modulation: increase of the glucose availability to cancer cells	[68]
	Brain-tropic, lung- tropic, bone-tropic or parental MDA-MB-231	CEMIP	Endothelial cells, microglia, astrocytes and neurons	Promoting adaptation to the brain microenvironment via vascular co-option	[69]
	MCF7-shXIST (breast cancer cells with low XIST expression (XIST^low^))	miR-503	Microglia	Microglia reprograming, immunity suppression	[70]
	B16-F10 (mouse melanoma cells)	MET	Bone marrow progenitor cells	Bone marrow cells’ education and mobilization	[71]
	Astrocytes	miR-19a	MDA-MB-231Br (brain metastatic breast cancer cells)	Recruitment of myeloid cells that enhance the brain metastatic tumor cells’ outgrowth	[72]

## Abbreviations

aSMase	Acid sphingomyelinase
BBB	Blood–brain barrier
BM	Brain metastases
CAFs	Cancer associated fibroblasts
CEMIP	Cell migration-inducing and hyaluronan-binding protein
CNS	Central nervous system
CSCs	Cancer stem cells
CSF	Cerebrospinal fluid
CTCs	Circulating tumor cells
DCs	Dendritic cells
DDS	Drug-delivery system
ECM	Extracellular matrix
EMT	Epithelial–mesenchymal transition
ESCRT	Endosomal sorting complexes required for transport
EVs	Extracellular vesicles
HSP	Heat shock protein
ILVs	Intraluminal vesicles
MSCs	Mesenchymal stem cells
MSP	Muscle-specific peptide
MVs	Microvesicles
MVB	Multivesicular body
NSCLC	Non-small cell lung cancer
nSMase	Neutral sphingomyelinase
PMN	Pre-metastatic niche
RVG	Rabies viral glycoprotein
SCLC	Small cell lung cancer
TDEs	Tumor derived exosomes
TME	Tumor microenvironment
TRAIL	Tumor necrosis factor-related apoptosis-inducing ligand
UC	Ultracentrifugation
VEGF	Vascular endothelial growth factor
